# Desmoplastic Reaction Associates with Prognosis and Adjuvant Chemotherapy Response in Colorectal Cancer: A Multicenter Retrospective Study

**DOI:** 10.1158/2767-9764.CRC-23-0073

**Published:** 2023-06-15

**Authors:** Qingru Hu, Yiting Wang, Su Yao, Yun Mao, Liu Liu, Zhenhui Li, Yonghe Chen, Shenyan Zhang, Qian Li, Yingnan Zhao, Xinjuan Fan, Yanfen Cui, Ke Zhao, Zaiyi Liu

**Affiliations:** 1 Department of Radiology, Guangdong Provincial People's Hospital (Guangdong Academy of Medical Sciences), Southern Medical University, Guangzhou, P.R. China.; 2 Guangdong Provincial Key Laboratory of Artificial Intelligence in Medical Image Analysis and Application, Guangzhou, P.R. China.; 3 The Second School of Clinical Medicine, Southern Medical University, Guangzhou, P.R. China.; 4 Department of Pathology, The Sixth Affiliated Hospital of Sun Yat-sen University, Guangzhou, P.R. China.; 5 Department of Pathology, Guangdong Provincial People's Hospital (Guangdong Academy of Medical Sciences), Southern Medical University, Guangzhou, P.R. China.; 6 Department of Radiology, The First Affiliated Hospital of Chongqing Medical University, Chongqing, P.R. China.; 7 Guangdong Cardiovascular Institute, Guangdong Provincial People's Hospital, Guangdong Academy of Medical Sciences, Guangzhou, P.R. China.; 8 Department of Radiology, The Third Affiliated Hospital of Kunming Medical University, Yunnan Cancer Hospital, Yunnan Cancer Center, Kunming, P.R. China.; 9 Department of Gastrointestinal Surgery, The Sixth Affiliated Hospital, Sun Yat-sen University, Guangzhou, P.R. China.; 10 School of Medicine, South China University of Technology, Guangzhou, P.R. China.; 11 School of Computer Science and Cyber Engineering, Guangzhou University, Guangzhou, P.R. China.; 12 Department of Radiology, Shanxi Cancer Hospital, Shanxi Medical University, Taiyuan, P.R. China.

## Abstract

Desmoplastic reaction (DR) is one of many tumor–host interactions and is associated with the overall survival (OS) of patients with colorectal cancer. However, the clinical significance of DR requires further study in large multicenter cohorts and its predictive value in adjuvant chemotherapy (ACT) response remains unclear. Here, a total of 2,225 patients with colorectal cancer from five independent institutions were divided into primary (*N* = 1,012 from two centers) and validation (*N* = 1,213 from three centers) cohorts. DR was classified as immature, middle, or mature depending on the presence of myxoid stroma and hyalinized collagen bundles at the invasive front of the primary tumor. OS among different subgroups were compared, and the correlations of DR type with tumor-infiltrating lymphocytes (TILs) within stroma, tumor stroma ratio (TSR), and Stroma AReactive Invasion Front Areas (SARIFA) were also analyzed. In the primary cohort, patients with mature DR had the highest 5-year survival rate. These findings were confirmed in validation cohort. In addition, for stage II colorectal cancer, patients classified as non-mature DR would benefit from ACT compared with surgery alone. Furthermore, immature and middle DR were more associated with high TSR, less distribution of TILs within stroma and positive SARIFA compared with mature. Taken together, these data suggest that DR is a robust-independent prognostic factor for patients with colorectal cancer. For patients with stage II colorectal cancer, non-mature DR could be a potential marker for recognizing high-risk patients who may benefit from ACT.

**Significance:**

DR has the potential to identify patients with high-risk colorectal cancer and predict the efficacy of adjuvant chemotherapy in patients with stage II colorectal cancer. Our findings support reporting DR types as additional pathologic parameters in clinical practice for more precise risk stratification.

## Introduction

Colorectal cancer remains the second leading cause of cancer-related mortality globally. Despite advances in diagnosis and treatment, the outcomes for patients with colorectal cancer persist highly variable, underscoring the complex and multifactorial nature of this disease (1). The existing tumor–node–metastasis (TNM) staging system, which primarily focuses on tumor cell–related factors, may not be sufficiently robust for accurate prognostic risk stratification, nor the decision management in the adjuvant chemotherapy (ACT) for patients with stage II colorectal cancer. In the context of standard primary cancer treatment, ACT was routinely recommended for patients with stage III–IV colorectal cancer and stage II patients with high-risk factors, but the individual clinical outcomes and treatment responses of patients with stage II colorectal cancer exhibit significant heterogeneity (2–4). Thus, there is an urgent need for complementary approaches that are potential of improving risk stratification and personalized treatment decisions for patients with colorectal cancer.

Over the past decades, numerous studies have demonstrated that the progression of colorectal cancer depends not only on the biological behavior of tumor cells but also on the interactions between these cells and tumor microenvironment (TME; refs. 5, 6). Distinct components in the tumor stroma were found providing important prognostic information in colorectal cancer and other epithelial malignancies (7–10). Among these stroma-related biomarkers, the desmoplastic reaction (DR) is an essential tumor-host response triggered by cancer-associated fibroblasts (CAFs) within TME (11). According to the existence of specific products of CAFs, myxoid stroma and hyalinized keloid-like collagen, DR was categorized into immature, middle, or mature type. Previous research has demonstrated the association of this classification system with colorectal cancer prognosis. Particularly, tumors with immature and intermediate DR were found to have worse prognoses compared with those with mature DR (12, 13). However, the prognostic value of DR requires further determination in a sizeable, multicenter sample and the significance of DR in terms of ACT is yet unclear.

Therefore, the primary objective of the current study was to investigate the validity of DR as a prognostic biomarker with large multicenter colorectal cancer patients. The exploratory aim was to evaluate the predictive value of DR for the postsurgical treatment regimen of patients with stage II colorectal cancer. In addition, this study sought to assess the correlation of DR with other elements in the TME.

## Materials and Methods

### Patients

According to the 8th American Joint Committee on Cancer TNM classification system, patients pathologically diagnosed as stage I–IV colorectal cancer and undergone radical primary tumor resection were enrolled from five independent institutions including Guangdong Provincial People's Hospital (GDPH), the Sixth Affiliated Hospital of Sun Yat-sen University (SYSU6), Shanxi Cancer Hospital (SXCH), the First Affiliated Hospital of Chongqing Medical University (CQMU1), and Yunnan Cancer Hospital (YNCH). Patients from GDPH and SYSU6 were included in the primary cohort, and others from SXCH, CQMU1, and YNCH were incorporated into the validation cohort. Exclusion criteria were as follows: (i) neoadjuvant therapy (radiotherapy, chemotherapy) before surgery; (ii) death within 30 days after surgery; (iii) follow-up information insufficient; (iv) hematoxylin and eosin (H&E)-stained whole-slide images (WSI) unavailable. Overall survival (OS), the period from diagnosis date to the time of death caused by any reason, was deemed as the event of interest. Disease-free survival (DFS), the time from surgery to disease relapse or patient death caused by disease progression, was deemed as the second event of interest. The survival status at the last follow-up date was recorded.

Clinicopathologic features of the primary and validation cohorts were recorded, which contained age at diagnosis, sex, tumor location (colon or rectum), TNM stage, grade [low (well/moderate differentiation) or high (poor differentiation)], carcinoembryonic antigen (CEA) level (cut-off value, 5 μg/L), microsatellite instability (MSI) status, lymphatic/vascular invasion (LVI), BRAF status, and peripheral nerve invasion (PNI). Our study had received the permission of the Institutional Review Boards in all these institutions mentioned above and the informed consent was waived.

### H&E and IHC Staining WSI Acquisition

H&E-stained slides of the most invasive part of primary tumor were used to determine the DR types. All these slides were scanned using digital WSI scanning systems (Aperio AT2, Leica; Aperio GT 450 Leica; MoticEasyScan Pro, Motic; KF-PRO-020, KFBIO; SQS-600P, TEKSQRAY; NanoZoomer S60) at 40 × magnification (resolution: 0.21–0.26 μm /pixel). Image annotation was carried out using the ImageScope software (ImageScope v12.4.3, Leica). Subsequently, 620 slides were selected for IHC. A series of steps were performed, deparaffinage, antigen retrieval solution (using 10 × concentrate solution, Novocastra, Leica) and primary [human anti-CD3 (Gene Tech, catalog no. GT200229) rabbit mAbs] and secondary (rabbit-anti-mouse IgG, Bond Refine Detection Kit, Leica) antibodies, according to the manufacturer's recommendations in a Ventana BenchMark automated staining system. Finally, the sections were incubated with 3,3-Diaminobenzidine, counterstained with hematoxylin, and mounted using special glue. To guarantee quality assurance, an internal positive control was utilized. The IHC-stained tissue sections were then captured utilizing a digital whole-slide scanning system (Aperio AT2, Leica) at 40 × magnification.

### The Evaluation Procedure of DR

DR was described as the formation of connective fiber tissue around tumor cells. One of the authors (Q. Hu) pathologically reviewed the primary tumors to evaluate the type of DR blinded to the patient's other clinical details. To assess the interobserver agreement, 200 samples were selected and evaluated independently by two co-authors (Y. Wang and S. Yao). According to the criteria described in previous study, the authors mentioned above underwent a rigorous training regimen of DR classification under the supervision of an experienced pathologist (S. Yao). Specifically, DR was classified into immature, middle, and mature groups on the basis of the appearance of myxoid stroma and keloid-like collagen in the invasive front. Myxoid stroma referred to an amorphous, mucous substance with mildly basophilic or amphophilic extracellular matrix. Keloid-like collagen appeared as a distinct hypocellular collagen bundle that was hyalinized, along with the brilliant eosinophilic hyalinization that was typically seen in keloid scars (11, 12). Findings in the submucosa and muscularis propria were also taken into consideration in classifying the DR pattern for investigating the prognostic significance of DR in stage I–IV colorectal cancer, which was different from the methodology adopted in prior investigations. In the WSI of the most invasive slide, a circle with a diameter of 500 μm was used to mark the specific components of different DR subtype in the invasive front of primary tumor. When myxoid stroma was detected and fulfilled the circle, DR was determined as immature type. Otherwise, it would be classified as middle type with keloid-like collagen shown in the stroma of invasive edge. In a tumor with neither myxoid stroma nor keloid-like collagen, DR was recognized as mature type, which consisted of fine mature multilayered collagen fiber. The agreements between two observers were evaluated using Cohen kappa and the overall weighted kappa was computed utilizing Light kappa.

### Assessment of Other Prognostic Factors in TME

#### Tumor-infiltrating Lymphocyte within Stroma

Tumor-infiltrating lymphocytes (TILs) reflect the host immune response triggered by the malignant process. The average density of CD3^+^ T cells within stroma was counted in the IHC slides of the most invasive part of primary tumor using a self-developed MATLAB software (R2020a, MathWorks; refs. 14–16).

#### Tumor Stroma Ratio

Tumor stroma ratio (TSR) is described as the stroma percentage in the tumor region. The stromal region was identified and the percentage was quantified through automated calculations utilizing a convolutional neural network reported in our previous studies (17). For statistical analysis, patients were divided into the stroma-low (TSR < 50%) group and the stroma-high (TSR ≥ 50%) group.

#### Stroma AReactive Invasion Front Areas

Stroma AReactive Invasion Front Areas (SARIFA) refers to the specific region where a tumor gland or a tumor cell cluster (≥5 cells) comes into direct contact with the surrounding adipose tissue in the invasion front (18, 19). Tumors that presented these characteristics were classified as SARIFA-positive and the others as SARIFA-negative. One of the authors (Q. Hu) assessed a subgroup of 620 cases blinded to other clinical data.

#### Tumor Budding

Tumor budding (TB) was described by the presence of a single cancer cell or clusters of fewer than five cancer cells in the invasive front according to previous research (20). The assessment of tumor budding was conducted in the subgroup of stage II patients with colorectal cancer by S. Yao. The procedure involved identifying solitary cancer cells or clusters of <5 cancer cells. Subsequently, a meticulous count of the budding foci was performed after selecting a microscopic field displaying noticeable budding at 20 × magnification. Tumors displaying <5, 5 to 9, and ≥10 budding foci were categorized as grade 1, grade 2, and grade 3, respectively.

### Assessment of DR in ACT

To assess the association between DR and ACT in stage II colorectal cancer, the prognosis of patients with stage II colorectal cancer in ACT group and surgery-only group was compared. The predominant chemotherapy regimens employed in this study were based on 5-fluorouracil, encompassing FOLFOX, FOLFIRI, XELOX, and similar variants. A minimum of a cycle of uninterrupted chemotherapy were administered to patients receiving this treatment.

### Statistical Analyses

Kaplan–Meier curves for OS and DFS were produced to demonstrate the distinctions in survival rates between patient groups, and *P* values were calculated by log-rank test. Continuous variables were compared by *t* tests. Categorical variables were compared by *χ*^2^ tests. *P* values of multiple comparisons were adjusted with Benjamini–Hochberg correction. *P* values less than 0.05 were considered significant statistically. Univariate and multivariate analysis were conducted using Cox proportional hazards regression models. Variables with *P* value less than 0.05 in univariate analyses were included in multivariate analyses. On the basis of the results of multivariate analyses, a nomogram was developed to estimate the OS of patients in primary and validation cohorts. The nomogram's ability to accurately predict OS was evaluated by C-index. Furthermore, the predictive accuracy of the nomogram was evaluated by boxplots with 1,000 × bootstrap resampling. R software (Version 4.1.2) was adopted for statistical analysis.

### Data Availability

The data produced in this investigation are presently unavailable to the public in consideration of the privacy concerning the involved patients. Nevertheless, data are available for collaborative analyses upon reasonable request by contacting the corresponding author.

## Results

### Clinicopathologic Characteristics

The numbers of patients in the primary and validation cohort were 1,012 and 1,213, respectively. The clinicopathologic characteristics were listed in Table 1. The median follow-up time of the primary and validation cohorts was 84.00 [95% confidence interval (CI), 67.44–92.40] months and 60.60 (95% CI, 48.24–89.88) months, respectively. Significant differences were found between the two cohorts on age, T status, N status, TNM stage, location, and tumor grade (all *P* < 0.05; Table 1). We analyzed the correlations between clinicopathologic factors and DR in patients with colorectal cancer in both primary and validation cohorts. It showed that non-mature DR was significantly associated with advanced T status (*P* < 0.01), lymph node metastasis (*P* < 0.01), tumor location (*P* < 0.01), and advanced TNM stage (*P* < 0.01; Supplementary Table S1). Higher T status was usually related more closely to less mature stroma (Supplementary Fig. S1A), and a similar trend was found in the relationship between DR and N status (Supplementary Fig. S1B).

**TABLE 1:** Patient demographics and clinicopathologic characteristics in two cohorts

	Primary cohort (*N* = 1,012)	Validation cohort (*N* = 1,213)	*P*
**Age**	61.9 ± 12.8	60.1 ± 12.6	<0.01
**Sex**			0.34
Male	601 (59.4%)	695 (57.3%)	
Female	411 (40.6%)	518 (42.7%)	
**T Status**			<0.01
1	32 (3.2%)	9 (0.7%)	
2	168 (16.6%)	78 (6.4%)	
3	723 (71.4%)	705 (58.1%)	
4	89 (8.8%)	421 (34.7%)	
**N Status**			<0.01
0	586 (57.9%)	815 (67.2%)	
1	283 (28.0%)	245 (20.2%)	
2	143 (14.1%)	153 (12.6%)	
**TNM Stage**			<0.01
I	168 (16.6%)	75 (6.2%)	
II	415 (41.0%)	735 (60.6%)	
III	409 (40.4%)	378 (31.2%)	
IV	20 (2.0%)	25 (2.1%)	
**Location**			0.04
Colon	523 (51.7%)	573 (47.2%)	
Rectum	489 (48.3%)	640 (52.8%)	
**CEA**			0.09
Normal	650 (64.2%)	752 (62.0%)	
Abnormal	301 (29.7%)	409 (33.7%)	
NA	61 (6.0%)	52 (4.3%)	
**Grade**			<0.01
High	88 (8.7%)	271 (22.3%)	
Low	906 (89.5%)	901 (74.3%)	
NA	18 (1.8%)	41 (3.4%)	
**MSI status**			1.00
MSI	88 (8.7%)	50 (4.1%)	
MSS	614 (60.7%)	352 (29.0%)	
NA	310 (30.6%)	811 (66.9%)	
**DR**			<0.01
Mature	507 (50.1%)	621 (51.2%)	
Middle	354 (35.0%)	294 (24.2%)	
Immature	151 (14.9%)	298 (24.6%)	

NOTE: CEA was available in 2,112 patients. MSI status was available in 1,104 patients and grade was available in 2,165 patients. Others were available in all patients.

Abbreviations: CEA, carcinoembryonic antigen; DR, desmoplastic reaction; NA, not available; MSI, microsatellite instability; MSS, microsatellite stability; TNM, tumor-node-metastasis.

### Prognostic Effect of DR Categorization

**FIGURE 1:**
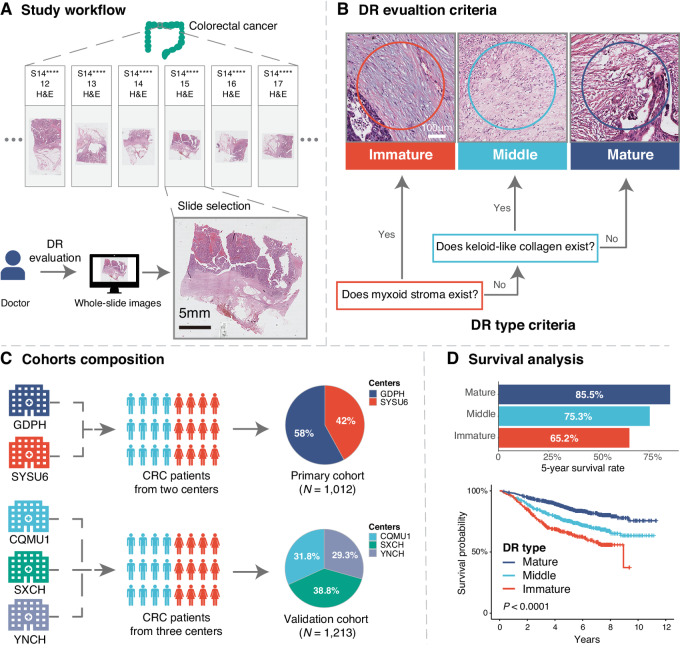
Study workflow. **A,** H&E-stained WSIs were used to assess the DR. **B,** Categorization of DR, DR in the reactive fibrous area at the extramural leading edge of the primary tumor were classified into three categories based on the presence or absence of myxoid stroma or keloid-like collagen. **C,** Cohort composition, primary cohort (from GDPH and SYSU6), validation cohort (from YNCH, SXCH, and CQMU1). **D,** The prognostic value of DR categorization was evaluated by Kaplan–Meier curves. CRC, colorectal cancer; DR, desmoplastic reaction; H&E, hematoxylin and eosin, WSIs, whole-slide images.

**FIGURE 2:**
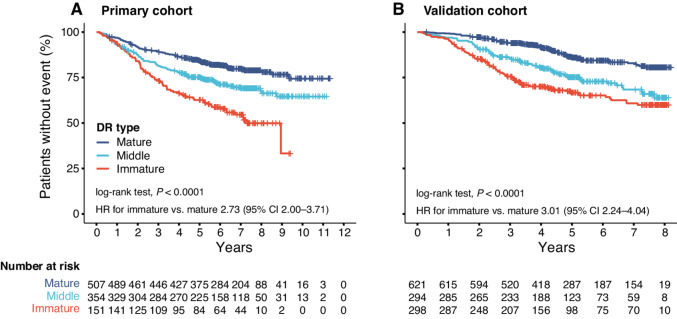
Prognostic significance of DR categorization for patients with colorectal cancer in the two cohorts. **A,** Primary cohort. **B,** Validation cohort. HR, hazard ratio; DR, desmoplastic reaction.

The overall workflow was presented in Fig. 1 and the representative images of mature/middle/immature DR were showed in Supplementary Fig. S2. The overall Light kappa value was 0.609 and kappa values of observer 1 versus 2, observer 2 versus 3, and observer 1 versus 3 were 0.612, 0.658, and 0.690, respectively, indicating good interobserver agreement (Supplementary Table S2). In the primary cohort, 507 (50.1%), 354 (35.0%), and 151(14.9%) patients were classified as colorectal cancer with mature, middle, and immature DR, respectively. Patients with mature DR had the highest survival rates. The 5-year OS rates of three DR groups were 85.5% (mature), 75.3% (middle), and 65.2% (immature), respectively (unadjusted HR for immature vs. mature 2.73; 95% CI, 2.00–3.71; *P* < 0.001; HR for middle vs. mature 1.62; 95% CI, 1.23–2.13, *P* = 0.001). Similar trend was observed in the validation cohort. (Fig. 2) Likewise, in the subgroup of 1,390 patients available for DFS data, the outcomes were comparable (Supplementary Fig. S3).

The prognostic association of DR with OS was maintained in multivariate analysis. In the primary cohort, DR was independent of age, CEA, and TNM stage, with immature and middle DR associated with worse OS. In the validation cohort, DR was independent of age, grade, and TNM stage (Table 2). Similar results were observed in the analysis based on DFS (Supplementary Table S3).

**TABLE 2:** Univariate and multivariate analyses in primary and validation cohorts

	Univariate Cox analysis				Multivariate Cox analysis			
	Primary cohort		Validation cohort		Primary cohort		Validation cohort	
	HR (95% CI)	*P*	HR (95% CI)	*P*	HR (95% CI)	*P*	HR (95% CI)	*P*
**TNM Stage**								
I	1		1		1		1	
II	1.96 (1.18–3.26)	<0.01	3.54 (1.29–9.66)	0.01	1.73 (1.00–2.98)	<0.05	2.32 (0.84–6.39)	0.10
III	4.45 (2.73–7.24)	<0.01	11.3 (4.19–30.7)	<0.01	3.45 (2.02–5.90)	<0.01	6.69 (2.44–18.4)	<0.01
IV	8.60 (4.14–17.9)	<0.01	48.9 (16.7–142)	<0.01	4.15 (1.86–9.29)	<0.01	39.82 (13.2–120)	<0.01
**Sex**								
Male	1		1		—		—	
Female	0.80 (0.62–1.02)	0.08	1.04 (0.81–1.33)	0.75	—		—	
**Age**	1.03 (1.02–1.05)	<0.01	1.02 (1.01–1.03)	<0.01	1.04 (1.02–1.05)	<0.01	1.03 (1.02–1.04)	<0.01
**Location**								
Colon	1		1		—		—	
Rectum	1.05 (0.83–1.34)	0.66	1.17 (0.91–1.49)	0.22	—		—	
**CEA**								
Normal	1		1		1		—	
Abnormal	2.63 (2.06–3.36)	<0.01	1.51 (1.17–1.94)	<0.01	1.94 (1.51–2.50)	<0.01	—	
**Grade**								
Low	1		1		—		1	
High	1.82 (1.27–2.59)	<0.01	1.69 (1.28–2.23)	<0.01	—		1.58 (1.19–2.10)	<0.01
**MSI status**								
MSI	1		1		—		—	
MSS	1.67 (1.00–2.79)	0.049	1.73 (0.53–5.67)	0.4	—		—	
**DR**								
Mature	1		1		1		1	
Middle	1.62 (1.23–2.13)	<0.01	2.09 (1.53–2.87)	<0.01	1.32 (0.98–1.76)	0.06	1.92 (1.38–2.65)	<0.01
Immature	2.73 (2.00–3.71)	<0.01	3.01 (2.24–4.04)	<0.01	1.84 (1.32–2.56)	<0.01	2.66 (1.94–3.64)	<0.01

NOTE: CEA was analyzed on the basis of 2,112 available patients. MSI status was available in 1,104 patients and grade was analyzed on the basis of 2,165 available patients. Others were analyzed on the basis of whole patients.

Abbreviations: 95%CI, 95% confidence interval; CEA, carcinoembryonic antigen; ; DR, desmoplastic reaction; HR, hazard ratio; MSI, microsatellite instability; MSS, microsatellite stability; TNM, tumor-node-metastasis.

To explore the influence of DR on prognosis in patients with different TNM stage, we performed subgroup analysis. The quantities of patients in each TNM stage were 243 (10.9%), 1,150 (51.6%), 787 (35.4), and 45 (2.1%), respectively. The maturity of DR was significantly correlated with OS in patients with stage II and III colorectal cancer. More mature DR was associated with longer OS time (both *P* < 0.0001, log-rank test). Yet similar trends were not observed in stage I and stage IV patients, nor did considerable differences exist (stage I *P* = 0.70, stage IV *P* = 0.81, respectively, log-rank test; Supplementary Fig. S4). In addition, DR remained a significant prognostic factor when stratified by clinicopathologic risk variables (Supplementary Fig. S5). For further evaluating the prognostic value of DR in patients with stage II colorectal cancer, we compared DR and TB with univariate analysis and found that the HR of the two variables was similar. In multivariate analysis, they are found to be independent of each other, and the adjusted HR remained comparable (Supplementary Table S4). In addition, middle and immature DR were significantly related to grade 2 and grade 3 TB (*P* < 0.01; Supplementary Table S5).

### Predictive Value of DR in Chemotherapy after Surgery

**FIGURE 3:**
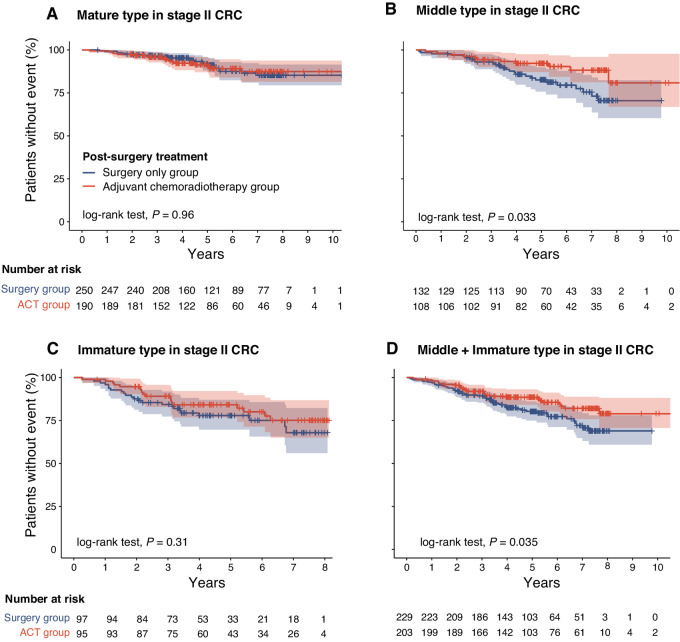
Prognostic significance of ACT in patients with stage II colorectal cancer with different DR. **A,** Mature. **B,** Middle. **C,** Immature. **D,** Middle and immature. ACT, adjuvant chemotherapy; CRC, colorectal cancer.

To investigate whether DR are favorable indicators for ACT in patients with stage II colorectal cancer, we compared the OS of patients assessed with different DR types between the ACT group and surgery-only group. ACT was performed in 45% of 872 stage II patients (*N* = 393). The clinicopathologic characteristics of patients in ACT and surgery only groups were presented in Supplementary Table S6. The Kaplan–Meier curves showed that the prognosis of patients in ACT group was significantly superior to surgery only group in patients assessed non-mature DR (5-year survival rates 88.5% vs. 80.3%, respectively, *P* = 0.035), which indicated that patients with non-mature DR are likely to benefit from ACT (Fig. 3). In the multivariate analyses, DR was independent of ACT (Supplementary Table S7). In addition, to further evaluate the predictive value of DR in adjuvant therapy, we performed additional analysis to examine the influence of several establish high-risk factors of stage II colorectal cancer, namely, BRAF status, LVI, PNI, TB, and MSI status on ACT efficacy in patient subgroups with relevant information and compared them with the results obtained from DR analysis (21). We found that while certain indicators, including BRAF status, PNI, TB, and LVI, showed positive trends toward better prognosis in the ACT group compared with the surgery-only group, there was no statistically significant difference in the prognosis of all high-risk subgroups (Supplementary Figs. S6–S8). This may potentially due to an inadequate sample size. Nevertheless, this still provides evidence to suggest that the value of DR in assessing the potential benefit of ACT in stage II patients is promising.

### Correlation of DR with Other Prognostic Factors in TME

**FIGURE 4:**
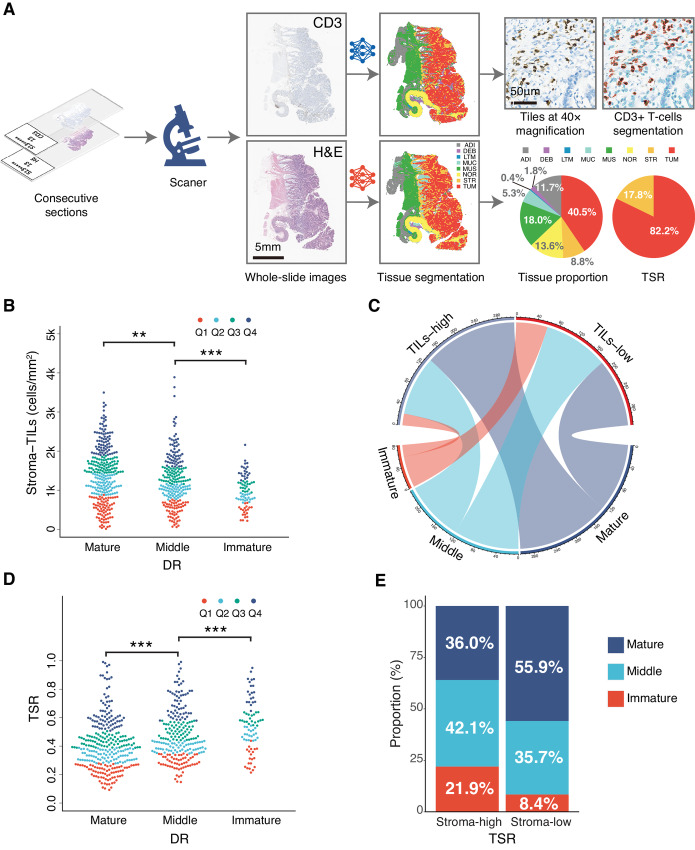
Correlation between DR and TILs, tumor stroma ratio. **A,** The workflow of TILs within stroma and TSR quantification. **B** and **C,** The correlation between DR and TILS within stroma. **D** and **E,** The correlation between DR and TSR. DR, desmoplastic reaction; TILs, tumor-infiltrating lymphocytes; TSR, tumor stroma ratio (Note: The *P* values of multiple comparisons in B and D were adjusted with Benjamini–Hochberg correction.).

To evaluate whether there could be a correlation of the DR with other prognostic factors in TME, additional analyses were performed. The automated quantification process of TILs and TSR was shown in Fig. 4A. The distribution of TILs within stroma versus DR was shown in Fig. 4B and C. Mature DR was correlated more closely with high TILs (>1,217 cells/mm^2^, the median density of TILs) within stroma while non-mature DR was related more closely to low TILs (≤1,217 cells/mm^2^) within stroma. The average density of mature, middle, and immature group was 1,399 cells/mm^2^, 1,229 cells/mm^2^, and 960 cells/mm^2^, respectively (mature vs. middle *P* < 0.01, middle vs. immature *P* < 0.001). Likewise, the proportion of non-mature DR was higher in stroma-high group than that in stroma-low group while the proportion of mature DR was the opposite (*P* < 0.0001; Fig. 4D and E). In addition, the proportion of non-mature DR was higher in SARIFA-positive group than that in SARIFA-negative group while the proportion of mature DR was the opposite (both *P* < 0.001; Supplementary Table S8).

### Performance of the DR-based Model

The model was constructed with five variables (age at diagnosis, TNM stage, CEA, grade, and DR) based on the findings of multivariate analysis. This model can be applied to estimate colorectal cancer patients’ 3- and 5-year postoperative survival rates. After that, C-indices were calculated to evaluate accuracy of the model in predicting patients’ 3- and 5-year OS (primary cohort, 0.736; 95% CI, 0.706–0.765; validation cohort, 0.729; 95% CI, 0.698–0.760, respectively; Supplementary Table S9). The model was presented as a nomogram as showed in Supplementary Fig. S9A. The box plots presented in Supplementary Fig. S9B and S9C showed that the model outperformed other parameters in terms of predictive ability in both the primary and validation cohorts.

## Discussion

There is significant heterogeneity in the outcomes and of patients with colorectal cancer, especially those determined I–II stages (22). The interactions between tumor cells and TME have been proven essential in tumor growth and shows great potential in the supplement of conventional tumor grading and staging approach. Among the established prognostic biomarkers in TME, DR aroused broad interest for the effectiveness of prognostic risk stratification and association with other stroma-related factors (23, 24). To the best of our knowledge, this multicenter study is by far the largest one to explore the prognostic value of DR on colorectal cancer. The findings from 2,225 patients provide strong evidence that myxoid stroma and keloid-like collagen indicated high risk of unfavorable outcomes and the prognostic nomogram based on DR showed great C-indices outperformed other parameters in terms of prognostic ability in both the primary and validation cohorts. Consistent with earlier studies addressing similar issues (23, 25, 26), our current study further explored the potential role of DR in predicting postsurgery chemotherapy efficacy.

Over the past decade, there has been a longstanding controversy over the postsurgery treatment of patients with stage II colorectal cancer (3, 27). Some studies pointed out that the lack of appropriate assessment criteria for cancer stroma may be largely to blame (28). Ao and colleagues demonstrated that CAFs derived from colorectal cancer tissue with immature-type DR overexpressed ADAM9s and stimulated cell proliferation and migration of colorectal cancer cell lines *in vitro*. In this regard, they supposed ADAM9-targeting antibody–drug conjugates may be a good drug candidate (29). However, predictive value of DR on ACT has been rarely reported in previous studies. In this research, we compared the outcomes of stage II patients who received and did not receive ACT in different DR subgroups and found that ACT could improve OS significantly in the non-mature group (*P* = 0.035). This indicated that middle and immature DR were predictive for ACT in stage II patients. Furthermore, in our data, the predictive value of DR on ACT is outperformed other known risk factors including BRAF status, PNI, MMR status, TB, and LVI. Thus, investigation of treatment regime based on DR holds great potential in the subsequent research. As far as we are aware, this is the first report that offers evidence suggesting that DR is potential of being involved in the treatment regime decision among patients with stage II colorectal cancer.

The interaction between DR and other stromal contents is also highly worthy of concentration for the tumor microenvironment is a complex collective of multiple elements. In this study, we found that immature DR was significantly associated with biomarkers that were indicative of unfavorable outcomes. In the correlation analysis between DR and TSR, immature DR appeared more frequently in the stroma-high group, which means, with the increase of TSR, the possibility of the formation of immature stroma also increased. When analyzing the association between DR and TILs within stroma, the distribution of TILs within stroma was found less distributed in the immature and middle group compared with mature. Similar findings were discovered in the investigation of the association between DR and SARIFA. Immature DR is strongly associated with positive SARIFA. Ueno and colleagues reported that pervasive distribution of myofibroblasts, a subtype of CAFs that promote tumor process by secreting enzymes that degrade the basement membrane and produce abundant extracellular matrix was observed in all tumors with immature DR while 47% of middle DR and 25% of mature DR (11). Highly invasive tumors may potentially tend to generate a stroma that is more favorable for tumor progression and metastasis through mechanism like this. In addition, fibro collagens and fibronectins were reported playing as physical barriers against immune infiltration, but there were also reports claimed that the fibroblast-produced matrix is probably required for inner tumor immune infiltration (30, 31). Our results help to explain the paradoxical effects of fibro components that mature fiber in stroma were in favor of immune infiltration while keloid-like collagen and myxoid stroma functioned as barriers.

It is worth noting that DR has advantages of strong prognostic and predictive value as well as simplified assessment procedures (32). Unlike other biomarkers that need complicated preprocessing for recognition, DR could be evaluated in H&E-stained slices directly thus available for regular clinical practice. Currently, several studies have successfully applied deep learning models to automate the identification, segmentation, and quantification of myxoid stroma with promising results (33, 34). As artificial intelligence technology continues to evolve, more accurate and objective DR classifiers are expected to offer significant benefits to clinicians and patients.

One of the limitations of this study is that the evaluation of DR was totally based on manual work and the bias caused by the subjectiveness is possible, but we do plan to achieve the automatic recognition and quantification in the future. Besides, this study is retrospective, and the results needed to be validated in prospective studies for routine clinical use.

Our study validated that DR is a robust prognostic biomarker in patients with colorectal cancer and suggested DR as a potential indicator in predicting the efficacy of ACT in patients with stage II colorectal cancer. Our findings suggest reporting DR types DR as additional pathologic parameters in clinical practice for more precise risk stratification and more individualized therapy for patients with colorectal cancer.

## Supplementary Material

Supplementary Table S1 Clinicopathological associations with desmoplastic reaction

Supplementary Table S2 Interobserver Agreement for DR

Supplementary Table S3 Uni- and multivariate analyses of DFS

Supplementary Table S4 Uni- and multivariate analyses in stage II CRC patients

Supplementary Table S5 Correlation between DR and TB

Supplementary Table S6 Patient clinicopathologic characteristics in ACT and surgery only groups

Supplementary Table S7 Multivariate analyses in stage II CRC patients

Supplementary Table S8 Correlation between DR and Stroma AReactive Invasion Front Areas

Supplementary Table S9 The discrimination performance in the two cohorts

Supplementary Figure S1 Correlation between DR and T status, N status

Supplementary Figure S2 Histological features of the desmoplastic reaction

Supplementary Figure S3 Correlation between DR and DFS

Supplementary Figure S4 Prognostic significance of DR categorization in each TNM stages of CRC patients

Supplementary Figure S5 Prognostic significance of DR categorization in different subgroups of CRC patients

Supplementary Figure S6 Predictive significance of other clinical risk factors on the ACT of stage II CRC

Supplementary Figure S7 Predictive significance of other TB on the ACT of stage II CRC

Supplementary Figure S8 Predictive significance of other MSI status on the ACT of stage II CRC

Supplementary Figure S9 Nomogram for OS and boxplots of C-indices in primary and validation cohorts
