# Supplementary material for: Desmoplastic Reaction Associates with Prognosis and Adjuvant Chemotherapy Response in Colorectal Cancer: A Multicenter Retrospective Study
Source: Cancer Res Commun. 2023 Jun 15;3(6):1057–66. doi: 10.1158/2767-9764.CRC-23-0073 (PMC10269709; doi:10.1158/2767-9764.CRC-23-0073)
Supplement: Supplementary Table S1 — Clinicopathological associations with desmoplastic reaction [file crc-23-0073-s01.pdf]

Supplementary Table S1. Clinicopathological associations with desmoplastic reaction

	Mature	Middle	Immature	P
**Age**	61.1 ± 12.3	61.3 ± 13.3	59.9 ± 13.0	0.16
**Sex**				0.83
Male	664 (58.9%)	374 (57.7%)	258 (57.5%)	
Female	464 (41.1%)	274 (42.3%)	191 (42.5%)	
**T Status**				<0.01
1	36 (3.2%)	5 (0.8%)	0 (0.0%)	
2	191 (16.9%)	49 (7.6%)	6 (1.3%)	
3	727 (64.5%)	430 (66.4%)	271 (60.4%)	
4	174 (15.4%)	164 (25.3%)	172 (38.3%)	
**N Status**				<0.01
0	812 (72.0%)	374 (57.7%)	215 (47.9%)	
1	213 (18.9%)	178 (27.5%)	137 (30.5%)	
2	103 (9.1%)	96 (14.8%)	97 (21.6%)	
**TNM Stage**				<0.01
I	194 (17.2%)	45 (6.9%)	4 (0.9%)	
II	615 (54.5%)	326 (50.3%)	209 (46.5%)	
III	308 (27.3%)	258 (39.8%)	221 (49.3%)	
IV	11 (1.0%)	19 (2.9%)	15 (3.3%)	
**Location**				<0.01
Colon	534 (47.3%)	358 (55.2%)	204 (45.4%)	
Rectum	594 (52.7%)	290 (44.8%)	245 (54.6%)	
**CEA**				0.42
Normal	758 (67.2%)	390 (60.2%)	254 (56.6%)	
Abnormal	316 (28.0%)	220 (34.0%)	174 (38.8%)	
NA	54 (4.8%)	38 (5.9%)	21 (4.7%)	
**Grade**				0.01
High	166 (14.7%)	115 (17.7%)	78 (17.4%)	
Low	925 (82.0%)	518 (79.9%)	364 (81.1%)	
NA	37 (3.3%)	15 (2.3%)	7 (1.6%)	

Note: CEA was analyzed based on 2112 available patients and grade was analyzed based on 2166 available patients. Others were analyzed on the basis of whole patients.

Abbreviations: TNM, tumor-node-metastasis; CEA, carcinoembryonic antigen; DR, desmoplastic reaction, NA, not available.
