# Supplementary material for: Desmoplastic Reaction Associates with Prognosis and Adjuvant Chemotherapy Response in Colorectal Cancer: A Multicenter Retrospective Study
Source: Cancer Res Commun. 2023 Jun 15;3(6):1057–66. doi: 10.1158/2767-9764.CRC-23-0073 (PMC10269709; doi:10.1158/2767-9764.CRC-23-0073)
Supplement: Supplementary Table S2 — Interobserver Agreement for DR [file crc-23-0073-s02.pdf]

Supplementary Table S2. Interobserver Agreement for DR

	Observer 1 vs 2	Observer 2 vs 3	Observer1 vs 3	Light's overall Kappa
**Kappa**	0.612	0.658	0.69	0.609

Abbreviations: DR, Desmoplastic reaction.
