# Supplementary material for: Desmoplastic Reaction Associates with Prognosis and Adjuvant Chemotherapy Response in Colorectal Cancer: A Multicenter Retrospective Study
Source: Cancer Res Commun. 2023 Jun 15;3(6):1057–66. doi: 10.1158/2767-9764.CRC-23-0073 (PMC10269709; doi:10.1158/2767-9764.CRC-23-0073)
Supplement: Supplementary Table S3 — Uni- and multivariate analyses of DFS [file crc-23-0073-s03.pdf]

Supplementary Table S3. Uni- and multivariate analyses of DFS

	Univariate Cox analysis		Multivariate Cox analysis	
	**HR (95%CI)**	**P**	**HR (95%CI)**	**P**
**Sex**				
Male	1			
Female	10.93 (0.76-1.12)	0.434	-	-
**Age**	1.03 (1.02-1.03)	<0.001	1.03 (1.02-1.04)	0.001
**Location**				
Colon	1		1	
Rectum	1.02 (0.84-1.24)	0.826	1.35(0.95–1.93)	0.1
**CEA**				
Normal	1		1	
Abnormal	2.09 (1.71-2.55)	<0.001	1.53 (1.25-1.88)	<0.001
**Grade**				
Low	1		1	
High	1.74 (1.31-2.31)	<0.001	1.40 (1.04-1.89)	0.024
**TNM Stage**				
I	1		1	
II	2.23 (1.44-3.44)	<0.001	1.96 (1.22-3.13)	0.005
III	5.19 (3.41-7.90)	<0.001	3.85 (2.41-6.15)	<0.001
IV	17.40 (10.26-29.5)	<0.001	14.72 (8.11-26.70)	<0.001
**DR**				
Mature	1		1	
Middle	1.75 (1.39-2.20)	<0.001	1.37 (1.07-1.75)	0.013
Immature	2.80 (2.21-3.55)	<0.001	2.08 (1.61-2.69)	<0.001

Note: DFS were available in 1390 patients. CEA was analyzed based on 1323 available patients. Grade was available in 1346 patients. Others were analyzed on the basis of all patients available of DFS.

Abbreviations: HR, hazard ratio; 95%CI, 95% confidence interval; TNM, tumor-node-metastasis; CEA, carcinoembryonic antigen; DR, desmoplastic reaction.
