# Supplementary material for: Desmoplastic Reaction Associates with Prognosis and Adjuvant Chemotherapy Response in Colorectal Cancer: A Multicenter Retrospective Study
Source: Cancer Res Commun. 2023 Jun 15;3(6):1057–66. doi: 10.1158/2767-9764.CRC-23-0073 (PMC10269709; doi:10.1158/2767-9764.CRC-23-0073)
Supplement: Supplementary Table S4 — Uni- and multivariate analyses in stage II CRC patients [file crc-23-0073-s04.pdf]

Supplementary Table S4. Uni- and multivariate analyses in stage II CRC patients

	Univariate Cox analysis		Multivariate Cox analysis	
	**HR(95%CI)**	**P**	**HR(95%CI)**	**P**
**TB**				
Grade 1	1		1	
Grade 2	1.49(1.00–2.22)	0.051	1.35(0.90–2.02)	0.15
Grade 3	2.26(1.50–3.39)	<0.001	1.98(1.31–3.00)	0.001
**DR**				
Mature	1		1	
Middle	1.39(0.98–1.99)	0.068	1.35(0.95–1.93)	0.1
Immature	2.23(1.54–3.23)	<0.001	1.98(1.36–2.90)	<0.001

Note: This was analyzed based on 872 stage II CRC patients.

Abbreviations: HR, hazard ratio; 95%CI, 95% confidence interval; TB, tumor budding; DR, desmoplastic reaction.
