# Supplementary material for: Desmoplastic Reaction Associates with Prognosis and Adjuvant Chemotherapy Response in Colorectal Cancer: A Multicenter Retrospective Study
Source: Cancer Res Commun. 2023 Jun 15;3(6):1057–66. doi: 10.1158/2767-9764.CRC-23-0073 (PMC10269709; doi:10.1158/2767-9764.CRC-23-0073)
Supplement: Supplementary Table S5 — Correlation between DR and TB [file crc-23-0073-s05.pdf]

Supplementary Table S5. Correlation between DR and TB.

	TB			
**DR**	**Grade 1**	**Grade 2**	**Grade 3**	**P**
**Mature**	506(82.3%)	75(12.2%)	34(5.5%)	<0.001
**Middle**	245(75.2%)	50(15.3%)	31(9.5%)	
**Immature**	125(59.8%)	48(23.0%)	36(17.2%)	

Note: This was analyzed based on 1150 stage II CRC.

Abbreviation: TB, tumor budding; DR, desmoplastic reaction.
