# Supplementary material for: Desmoplastic Reaction Associates with Prognosis and Adjuvant Chemotherapy Response in Colorectal Cancer: A Multicenter Retrospective Study
Source: Cancer Res Commun. 2023 Jun 15;3(6):1057–66. doi: 10.1158/2767-9764.CRC-23-0073 (PMC10269709; doi:10.1158/2767-9764.CRC-23-0073)
Supplement: Supplementary Table S6 — Patient clinicopathologic characteristics in ACT and surgery only groups [file crc-23-0073-s06.pdf]

Supplementary Table S6. Patient clinicopathologic characteristics in ACT and surgery only groups

	Surgery only group (N = 479)	ACT group (N = 393)	P
**Age**	65.6 ± 11.7	56.5 ± 11.7	<0.001
**Sex**			0.216
Male	279(58.2%)	246(626%)	
Female	200(41.8%)	147(37.4%)	
**Location**			0.924
Colon	235(49.1%)	195(49.6%)	
Rectum	244(50.9%)	198(50.4%)	
**CEA**			0.791
Normal	312(65.1%)	258(65.6%)	
Abnormal	132(27.6%)	115(29.3%)	
NA	35(7.3%)	20 (5.1%)	
**Grade**			0.084
High	58(12.1%)	64(16.3%)	
Low	413(86.2%)	319(81.2%)	
NA	8(1.7%)	10(2.5%)	
**DR**			
Mature	250(52.2%)	190(48.3%)	0.343
Middle	132(27.6%)	108(27.5%)	
Immature	97(20.3%)	95(24.2%)	

Note: CEA was available in 817 patients and grade was available in 854 patients. Others were available in all 872 patients with treatment information.

Abbreviation: CEA, carcinoembryonic antigen; DR, desmoplastic reaction; NA, not available.
