# Supplementary material for: Desmoplastic Reaction Associates with Prognosis and Adjuvant Chemotherapy Response in Colorectal Cancer: A Multicenter Retrospective Study
Source: Cancer Res Commun. 2023 Jun 15;3(6):1057–66. doi: 10.1158/2767-9764.CRC-23-0073 (PMC10269709; doi:10.1158/2767-9764.CRC-23-0073)
Supplement: Supplementary Table S7 — Multivariate analyses in stage II CRC patients [file crc-23-0073-s07.pdf]

Supplementary Table S7. Multivariate analyses in stage II CRC patients

	HR(95%CI)	P
**Treatment**		
Surgery only	1	
ACT	0.71(0.49–1.03)	0.074
**DR**		
Mature	1	
Middle	1.05(0.49–2.25)	0.9
Immature	2.12(1.09–4.12)	0.026

Note: This was analyzed based on 872 stage II CRC patients with treatment information.

Abbreviation: HR, hazard ratio; 95%CI, 95% confidence interval; ACT, adjuvant chemotherapy; DR, desmoplastic reaction.
