# Supplementary material for: Desmoplastic Reaction Associates with Prognosis and Adjuvant Chemotherapy Response in Colorectal Cancer: A Multicenter Retrospective Study
Source: Cancer Res Commun. 2023 Jun 15;3(6):1057–66. doi: 10.1158/2767-9764.CRC-23-0073 (PMC10269709; doi:10.1158/2767-9764.CRC-23-0073)
Supplement: Supplementary Table S8 — Correlation between DR and Stroma AReactive Invasion Front Areas [file crc-23-0073-s08.pdf]

Supplementary Table S8. Correlation between DR and Stroma AReactive Invasion Front Areas.

	SARIFA		
**DR**	**Negative**	**Positive**	**P**
**Mature**	291(95.7%)	13(4.3%)	<0.001
**Middle**	187(79.6%)	48(20.4%)	
**Immature**	25(30.9%)	56(69.1%)	

Note: This was analyzed based on 620 patients available for SARIFA status.

Abbreviation: SARIFA, Stroma AReactive Invasion Front Areas; DR, desmoplastic reaction.
