# Supplementary material for: Desmoplastic Reaction Associates with Prognosis and Adjuvant Chemotherapy Response in Colorectal Cancer: A Multicenter Retrospective Study
Source: Cancer Res Commun. 2023 Jun 15;3(6):1057–66. doi: 10.1158/2767-9764.CRC-23-0073 (PMC10269709; doi:10.1158/2767-9764.CRC-23-0073)
Supplement: Supplementary Table S9 — The discrimination performance in the two cohorts [file crc-23-0073-s09.pdf]

Supplementary Table S9. The discrimination performance in the two cohorts.

	**Discovery cohort**	**Validation cohort**
	C-index (95% CI)	C-index (95% CI)
**Age**	0.610 (0.574–0.646)	0.571 (0.534–0.608)
**TNM stage**	0.652 (0.622–0.681)	0.694 (0.663–0.724)
**CEA**	0.626 (0.596–0.657)	0.553 (0.521–0.584)
**Grade**	0.536 (0.513–0.558)	0.548 (0.518–0.578)
**DR**	0.596 (0.564–0.628)	0.644 (0.612–0.677)
**Model**	0.736 (0.706–0.765)	0.729 (0.698–0.760)

Note: C-index of CEA was calculated based on 2112 available patients and C-index of Grade was calculated based on 2165 available patients. Others were calculated on the basis of whole patients.

Abbreviations: C-index, Harrell's concordance index; CI, confidence interval; TNM, tumor-node-metastasis; CEA; DR, desmoplastic reaction.
