# Supplementary material for: Desmoplastic Reaction Associates with Prognosis and Adjuvant Chemotherapy Response in Colorectal Cancer: A Multicenter Retrospective Study
Source: Cancer Res Commun. 2023 Jun 15;3(6):1057–66. doi: 10.1158/2767-9764.CRC-23-0073 (PMC10269709; doi:10.1158/2767-9764.CRC-23-0073)
Supplement: Supplementary Figure S1 — Correlation between DR and T status, N status [file crc-23-0073-s10.pdf]

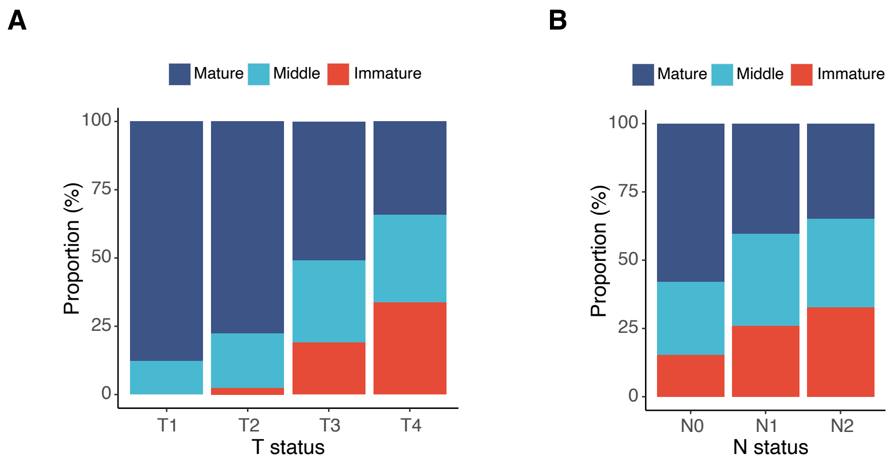

Supplementary Figure S1. Correlation between DR and T status, N status. (A) The proportion of each DR types in different T status. (B) The proportion of each DR types in different N statuses.
