# Supplementary material for: Desmoplastic Reaction Associates with Prognosis and Adjuvant Chemotherapy Response in Colorectal Cancer: A Multicenter Retrospective Study
Source: Cancer Res Commun. 2023 Jun 15;3(6):1057–66. doi: 10.1158/2767-9764.CRC-23-0073 (PMC10269709; doi:10.1158/2767-9764.CRC-23-0073)
Supplement: Supplementary Figure S2 — Histological features of the desmoplastic reaction [file crc-23-0073-s11.pdf]

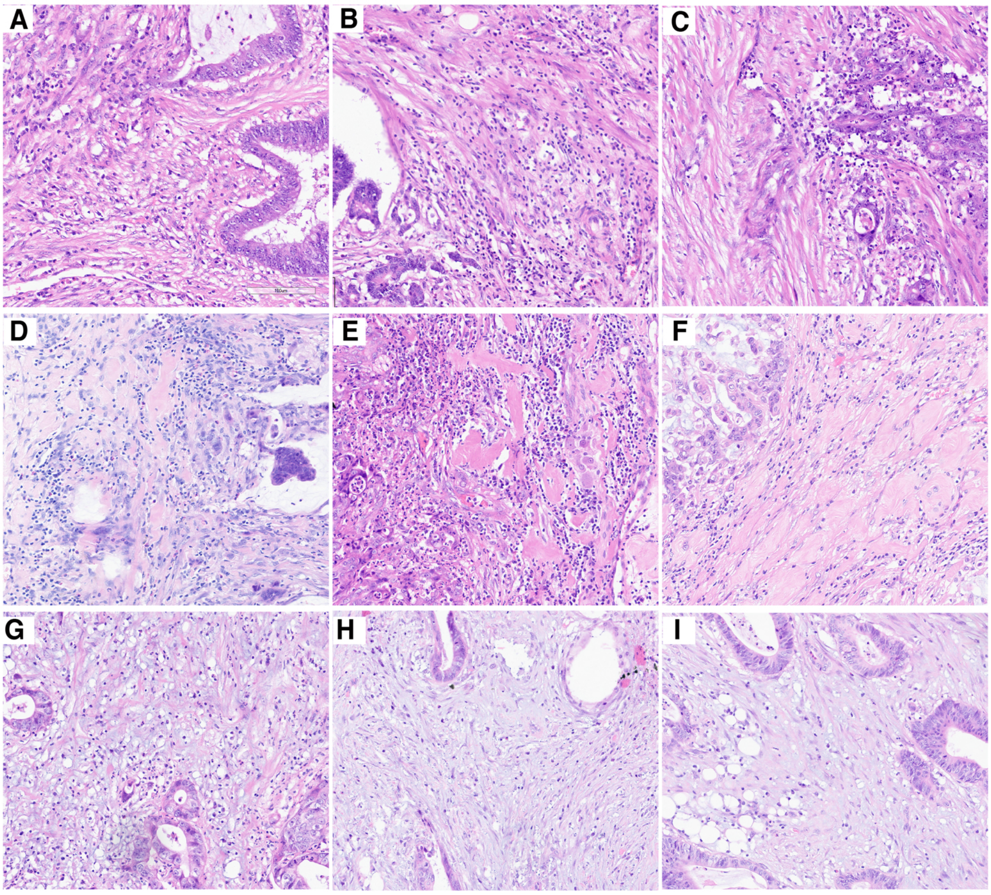

Supplementary Figure S2. Histological features of the desmoplastic reaction. (A-C) Fibrotic stroma without myxoid stroma and keloid-like collagen, typically characterized by fine fibers stratified into multilayers (mature type). (D-F) Fibrotic stroma containing keloid-like collagen, i.e., broad bands of collagen with bright eosinophilic hyalinization, similar to those observed in a keloid (middle type). (G-I) Abundant extracellular matrix substance accumulated at the leading edge of the tumor to form myxoid stroma (immature type).
