# Supplementary material for: Desmoplastic Reaction Associates with Prognosis and Adjuvant Chemotherapy Response in Colorectal Cancer: A Multicenter Retrospective Study
Source: Cancer Res Commun. 2023 Jun 15;3(6):1057–66. doi: 10.1158/2767-9764.CRC-23-0073 (PMC10269709; doi:10.1158/2767-9764.CRC-23-0073)
Supplement: Supplementary Figure S3 — Correlation between DR and DFS [file crc-23-0073-s12.pdf]

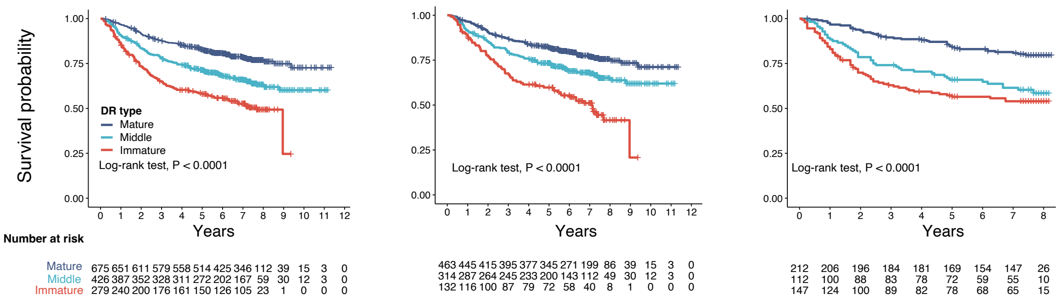

Supplementary Figure S3. Correlation between DR and DFS: (A)Whole cohort. (B) Primary cohort. (C) Validation cohort.
