# Supplementary material for: Desmoplastic Reaction Associates with Prognosis and Adjuvant Chemotherapy Response in Colorectal Cancer: A Multicenter Retrospective Study
Source: Cancer Res Commun. 2023 Jun 15;3(6):1057–66. doi: 10.1158/2767-9764.CRC-23-0073 (PMC10269709; doi:10.1158/2767-9764.CRC-23-0073)
Supplement: Supplementary Figure S4 — Prognostic significance of DR categorization in each TNM stages of CRC patients [file crc-23-0073-s13.pdf]

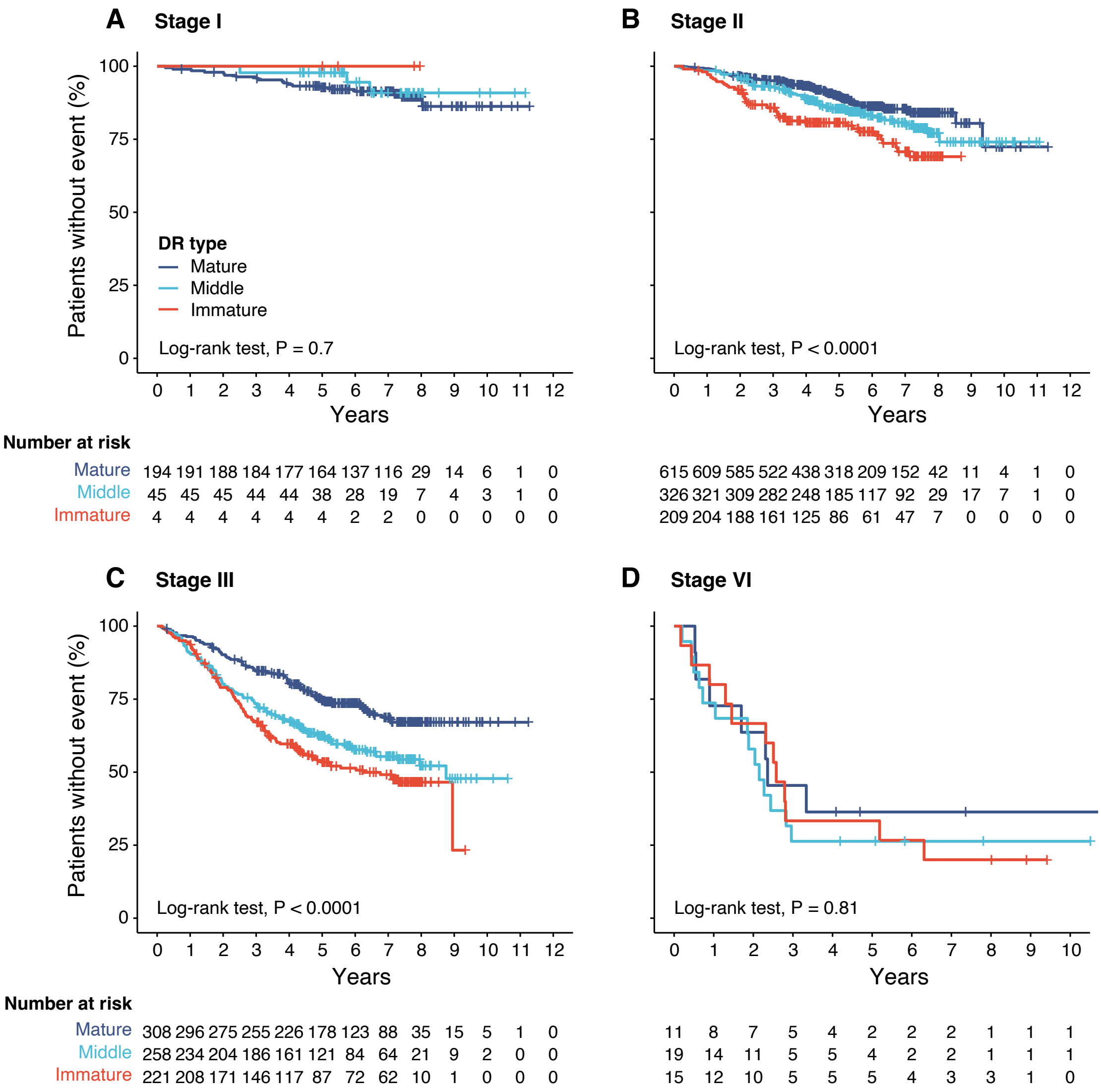

Supplementary Figure S4. Prognostic significance of DR categorization in each TNM stages of CRC patients. (A) stage I. (B) stage II. (C) stage III. (D) stage IV. Mature DR was associated with longer OS in stage II, and III CRC patients (both P < 0.0001, log-rank test). CRC, colorectal cancer; TNM, tumor-node-metastasis; DR, desmoplastic reaction.
