# Supplementary material for: Desmoplastic Reaction Associates with Prognosis and Adjuvant Chemotherapy Response in Colorectal Cancer: A Multicenter Retrospective Study
Source: Cancer Res Commun. 2023 Jun 15;3(6):1057–66. doi: 10.1158/2767-9764.CRC-23-0073 (PMC10269709; doi:10.1158/2767-9764.CRC-23-0073)
Supplement: Supplementary Figure S5 — Prognostic significance of DR categorization in different subgroups of CRC patients [file crc-23-0073-s14.pdf]

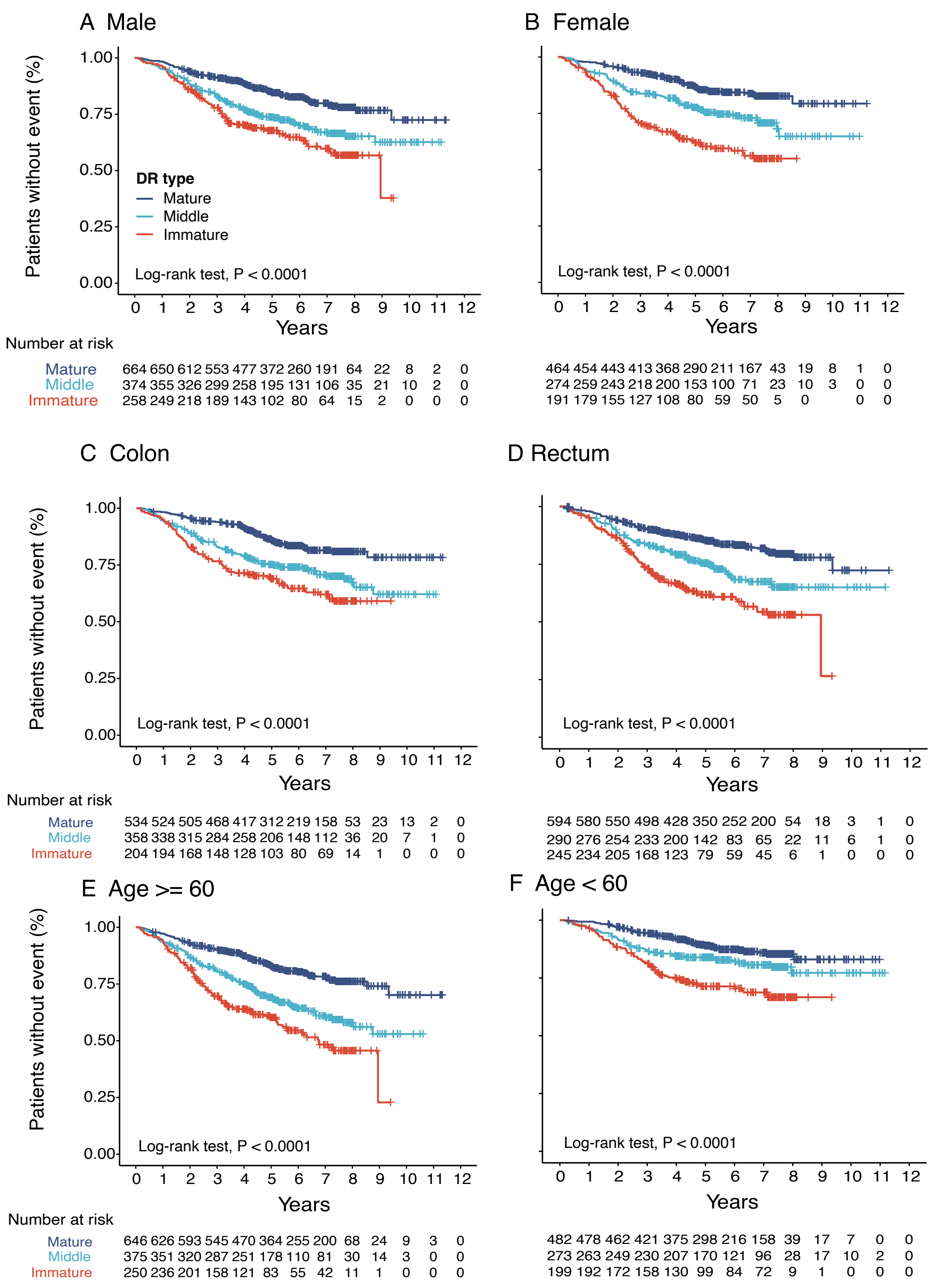

Supplementary Figure S5. Prognostic significance of DR categorization in different subgroups of CRC patients. (A–B) sex. (C–D) location of tumor. (E–F) age (all P<0.0001, log-rank test). DR, Desmoplastic reaction.
