# Supplementary material for: Desmoplastic Reaction Associates with Prognosis and Adjuvant Chemotherapy Response in Colorectal Cancer: A Multicenter Retrospective Study
Source: Cancer Res Commun. 2023 Jun 15;3(6):1057–66. doi: 10.1158/2767-9764.CRC-23-0073 (PMC10269709; doi:10.1158/2767-9764.CRC-23-0073)
Supplement: Supplementary Figure S6 — Predictive significance of other clinical risk factors on the ACT of stage II CRC [file crc-23-0073-s15.pdf]

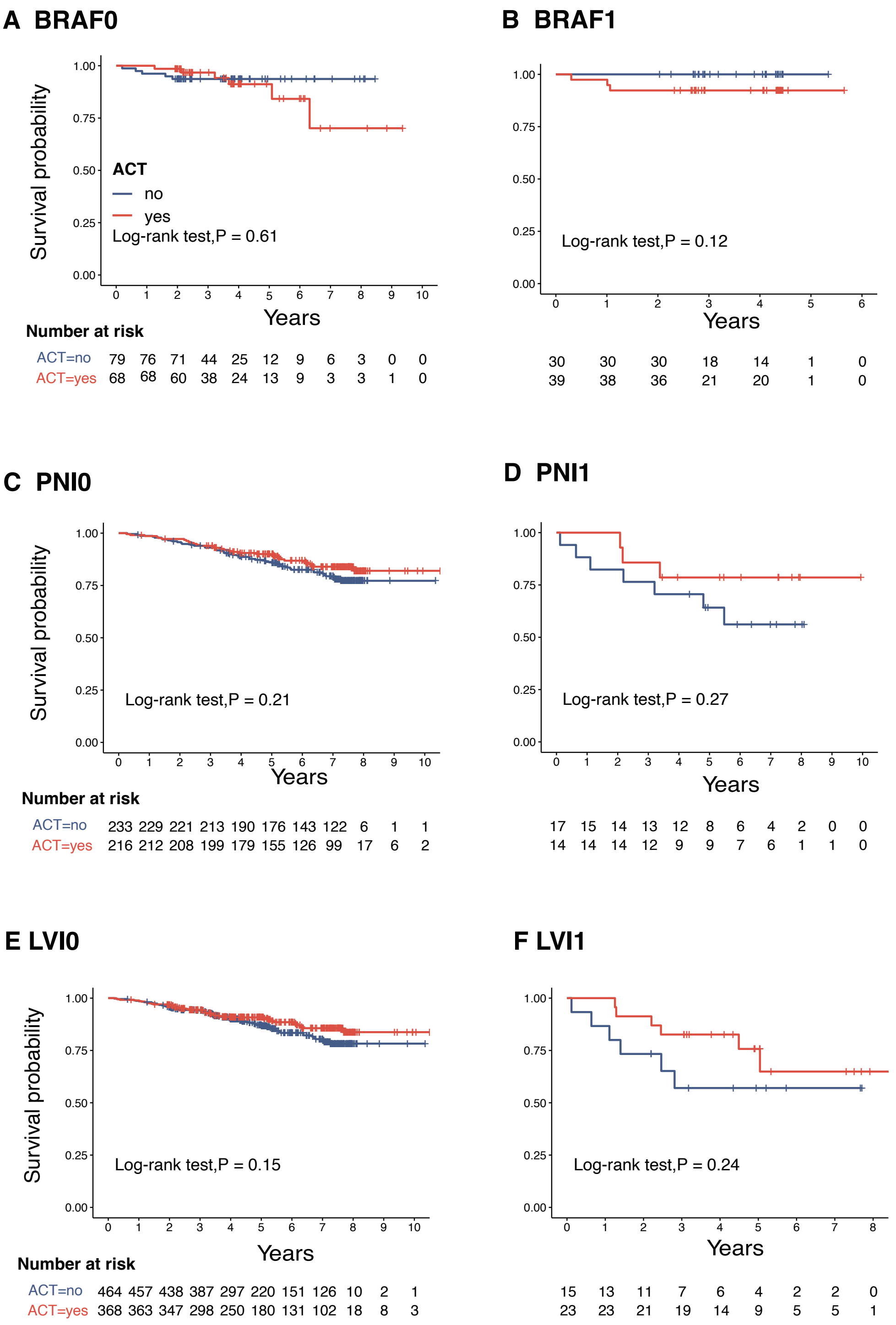

Supplementary Figure S6. Predictive significance of other clinical risk factors on the ACT of stage II CRC. (A–B) BRAF status. (C–D) PNI. (E–F) LVI. ACT, adjuvant chemotherapy. PNI, peripheral nerve invasion. LVI, lymphatic/vascular invasion.
