# Supplementary material for: Desmoplastic Reaction Associates with Prognosis and Adjuvant Chemotherapy Response in Colorectal Cancer: A Multicenter Retrospective Study
Source: Cancer Res Commun. 2023 Jun 15;3(6):1057–66. doi: 10.1158/2767-9764.CRC-23-0073 (PMC10269709; doi:10.1158/2767-9764.CRC-23-0073)
Supplement: Supplementary Figure S7 — Predictive significance of other TB on the ACT of stage II CRC [file crc-23-0073-s16.pdf]

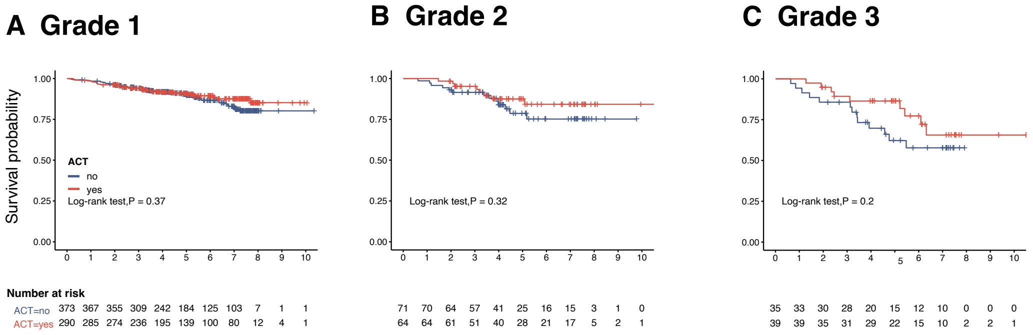

Supplementary Figure S7. Predictive significance of other TB on the ACT of stage II CRC. (A) Grade 1. (B) Grade 2. (C) Grade 3. ACT, adjuvant chemotherapy. TB, tumor budding.
