# Supplementary material for: Desmoplastic Reaction Associates with Prognosis and Adjuvant Chemotherapy Response in Colorectal Cancer: A Multicenter Retrospective Study
Source: Cancer Res Commun. 2023 Jun 15;3(6):1057–66. doi: 10.1158/2767-9764.CRC-23-0073 (PMC10269709; doi:10.1158/2767-9764.CRC-23-0073)
Supplement: Supplementary Figure S8 — Predictive significance of other MSI status on the ACT of stage II CRC [file crc-23-0073-s17.pdf]

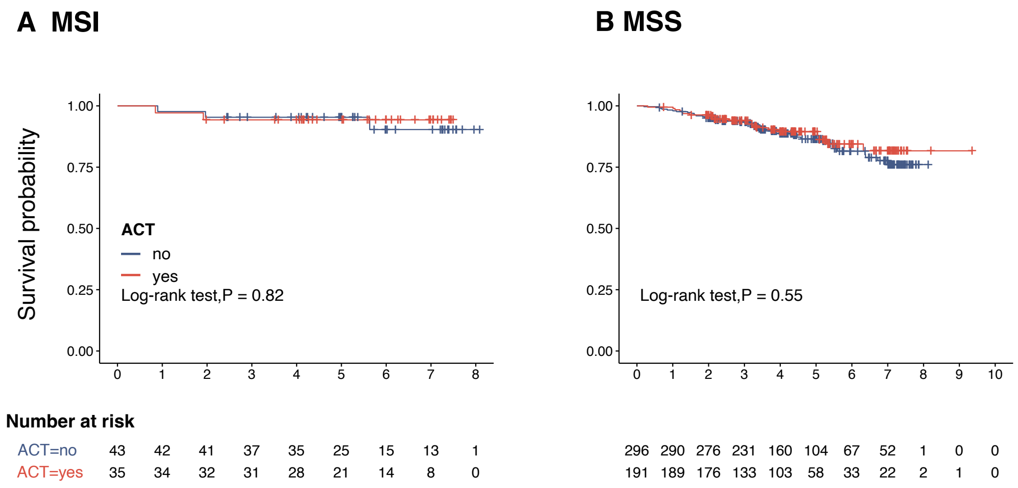

Supplementary Figure S8. Predictive significance of other MSI status on the ACT of stage II CRC.

(A) MSI. (B) MSS. ACT, adjuvant chemotherapy. MSI, microsatellite instability; MSS, microsatellite stability.
