# Supplementary material for: Desmoplastic Reaction Associates with Prognosis and Adjuvant Chemotherapy Response in Colorectal Cancer: A Multicenter Retrospective Study
Source: Cancer Res Commun. 2023 Jun 15;3(6):1057–66. doi: 10.1158/2767-9764.CRC-23-0073 (PMC10269709; doi:10.1158/2767-9764.CRC-23-0073)
Supplement: Supplementary Figure S9 — Nomogram for OS and boxplots of C-indices in primary and validation cohorts [file crc-23-0073-s18.pdf]

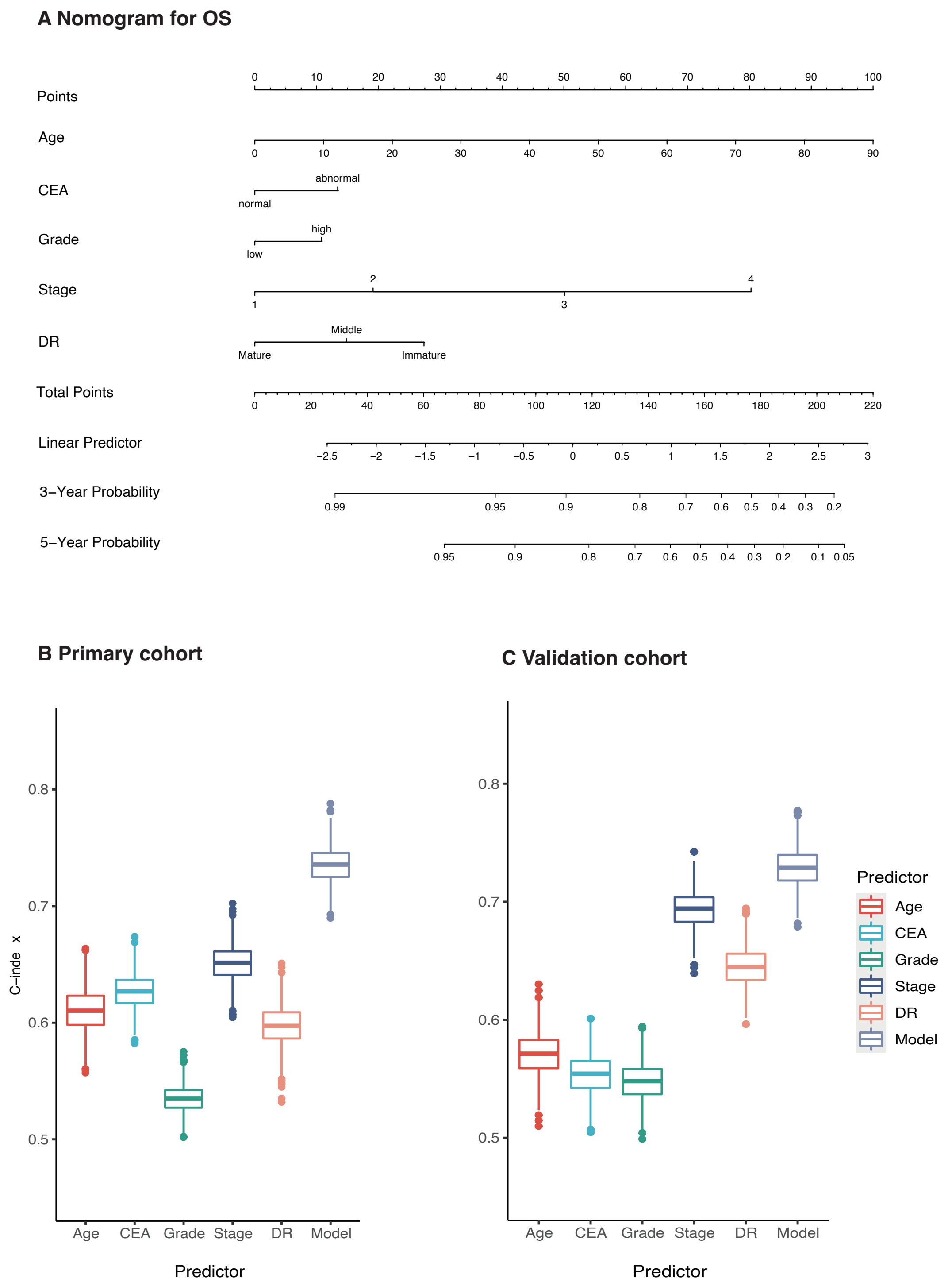

Supplementary Figure S9. Nomogram for OS and boxplots of C-indices in primary and validation cohorts. (A) Nomogram for OS. (B) Boxplots of C-indices in primary cohort. (C) Boxplots of C-indices in validation cohort. DR, desmoplastic reaction; OS, overall survival; CEA, carcinoembryonic antigen.
